# Iron phthalocyanine-sensitized magnetic catalysts for BPA photodegradation

**DOI:** 10.1038/s41598-020-61980-6

**Published:** 2020-03-25

**Authors:** Mariana Neamtu, Claudia Nadejde, Loredana Brinza, Oana Dragos, Daniela Gherghel, Andrea Paul

**Affiliations:** 1 Alexandru Ioan Cuza University of Iasi, Institute for Interdisciplinary Research – Science Research Department, Lascar Catargi Str. 54, 700107 Iasi, Romania; 2 National Institute of Research and Development for Technical Physics, Dimitrie Mangeron Bd. 47, 700050 Iasi, Romania; 3 Institute of Biological Research Iasi, Experimental and Applied Biology Department, Lascar Catargi Str. 47, 700107 Iasi, Romania; 4 Bundesanstalt für Materialforschung und -prüfung (BAM), Unter den Eichen 87, 12205 Berlin, Germany

**Keywords:** Environmental sciences, Chemistry, Materials science

## Abstract

The catalytic behavior of iron phthalocyanine (FePc)-sensitized magnetic nanocatalysts was evaluated for their application in the oxidative treatment of Bisphenol A (BPA) under mild environmental conditions. Two types of FePc (Fe(II)Pc and Fe(III)Pc), which are highly photosensitive compounds, were immobilized on the surface of functionalized magnetite. The nanomaterials were characterized by high resolution transmission electron microscopy (HR-TEM), X-ray diffraction (XRD), Fourier transform infrared spectroscopy (FTIR) and thermogravimetric analyses (TGA). The generation of singlet oxygen by nanomaterials was also investigated. In the presence of UVA light exposure (365 nm) and 15 mM H_2_O_2_, the M@Fe(III)Pc photocatalyst gave the best results; for a catalyst concentration of 2.0 g L ^− 1^, around 60% BPA was removed after 120 min of reaction. These experimental conditions were further tested under natural solar light exposure, for which also M@Fe(III)Pc exhibited enhanced oxidative catalytic activity, being able to remove 83% of BPA in solution. The water samples were less cytotoxic after treatment, this being confirmed by the MCF-7 cell viability assay.

## Introduction

Emerging recalcitrant organic pollutants received extensive attention over the years, since they are difficult to remove using conventional technologies. Continuous efforts are made to find improved solutions for fighting against and for diminishing environmental pollution. Endocrine disruptive chemicals (EDC) are a class of hazardous persistent pollutants exhibiting numerous harmful effects, able to induce subtle and irreversible changes in living organisms, even in concentrations of few parts per billion^1,2^. Bisphenol A (BPA) is an EDC that remains a major water contaminant, since its production in the 1950s. Since then, BPA become a well-established model compound for persistent micropollutants. Although found in low concentration in water, its removal proved to be unsuccessful using conventional water treatment approaches, such as chlorination and ozonation, processes that also exhibit several draw-backs^3^. Promising alternatives, such as photocatalysis^1,4–6^, were found to be very efficient and versatile. For instance, photocatalysis involving dye-sensitization represents an eco-friendly approach with great potential for very efficient removal of persistent hazardous pollutants from waters. Singlet oxygen, a reactive oxygen species (ROS), has proved to be an extremely effective agent in the oxidation of emerging pollutants, due to its high oxidation ability.

Organic phthalocyanines (Pcs) are the synthetic analogues of natural porphyrine compounds^7^. Discovered in 1928, Pcs, possessing a two-dimensional geometry and a ring system consisting of 18 π-electrons, are composed of four isindole units linked by nitrogen atoms. Due to the large, aromatic, macrocyclic structure of Pc, a variety of metals (Me), such as Fe^8–10^, Zn^11,12^, Cu^13,14^, Mn, Co, Ni^15,16^, Si^17^, Ru^18^, can be bonded inside the cycle of Pc, substituting the two hydrogen atoms. These metals can tune the fine structure of the new functionalized compound as well as its physical and chemical properties. Pcs and their metalo-derivates exhibit numerous remarkable features such as excellent chemical and thermal stability, high response to light exposure in a large range from UV to near infrared region of the light spectrum, etc. Moreover, their ability to generate singlet oxygen, makes them ideal photosensitizers. Thus, they found applicability in various fields. Recent advances in phthalocyanine-based functional materials are presented in a very comprehensive review by Bian and Jiang^19^. A detailed review of Pc synthesis and application as catalysts is given by Sorokin^20^. Among application as catalysts, Pcs have been received special attention as their efficiency can potentially be increased by the presence of Fe, which may play an important role due to the redox dynamic structure of the Fe(II)/Fe(III) in oxygen reduction reaction (ORR) and photocatalysis. In literature, a limited number of studies were found about the potential of single–atom catalysts of the iron phthalocyanine (FePc) mechanism for the hydrogen peroxide activated catalytic decomposition of recalcitrant organic pollutants.

In comparison with homogeneous photocatalysis, heterogeneous photocatalysis presents significant advantages; in the latter mentioned procedures, the FePcs can be fixed onto solid supports, such as magnetic substrates, which allow easy retrieval of the material from the environment without causing additional pollution; this also provides the possibility of material reuse in subsequent water treatment cycles. Magnetite (Fe_3_O_4_) is a popular choice among magnetic carriers, being recognized as an eco-friendly material with numerous important advantages and extensive applications. The incorporation of Fe_3_O_4_ particles in polymers provides material stability, protection against oxidation, nanoparticle dispersion and possibility for further functionalization. Based on the versatile reactivity of phthalocyanine with metal and outstanding physical properties of MePc, introducing the phthalocyanine to Fe_3_O_4_ nanoparticles may result into a new hybrid material whose properties can be different not only from the single polymer but also from the Fe_3_O_4_, due to the synergistic effects derived from the interaction between them. The few reported data in the literature demonstrates that such interaction generally leads to improved photophysical and photochemical properties of MePc- Fe_3_O_4_ systems^8,10,21^.

Hence, in this study, we report the synthesis and photocatalytic behavior of FePc-sensitized magnetic nanoparticles for the photodegradation of BPA in solution. The combination of advanced oxidation processes with light-responsive FePc immobilized on magnetic nanoparticles was investigated for efficient BPA photodegradation at near-neutral pH, using different catalyst dosages and H_2_O_2_ concentrations, under different light sources. The obtained magnetic nanocatalysts were easy recoverable and recyclable, possessing good photocatalytic activity, especially under visible light photoactivation.

## Results and discussion

### Photocatalyst characterization

**Figure 1:**
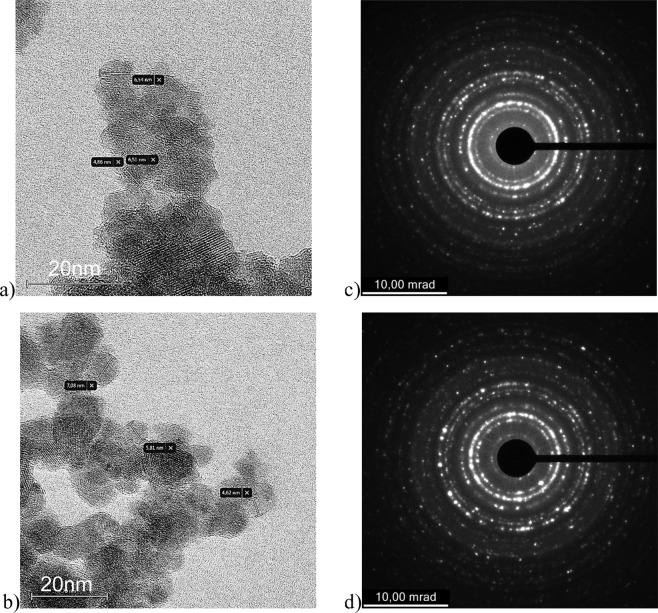
HR-TEM images of (**a**) M@Fe(II)Pc, (**b**) M@Fe(III)Pc and the corresponding SAED patterns of (**c**) M@Fe(II)Pc and (**d**) M@Fe(III)Pc.

The high-resolution transmission electron microscopy analyses were used in order to reveal the morphological properties of the catalysts. HR-TEM images (Fig. 1a,b, left column) confirmed the detailed morphology and nanoscale dimension of the magnetic photocatalysts; the quasi-spherical shape of the nanoparticles, with diameters up to 15 nm, were identified in each sample, which is the typical result following the application of co-precipitation method for magnetic nanoparticle synthesis. A thin coating of *ca*. 1 nm was identified to surround the magnetic cores. The Selected Aria Electron Difraction (SAED) images (Fig. 1c,d, right column) confirmed that the diffraction rings belong to the magnetite crystalline phase, while the coating of the magnetite cores with organic shell is evidenced by the hollow rings.

**Figure 2:**
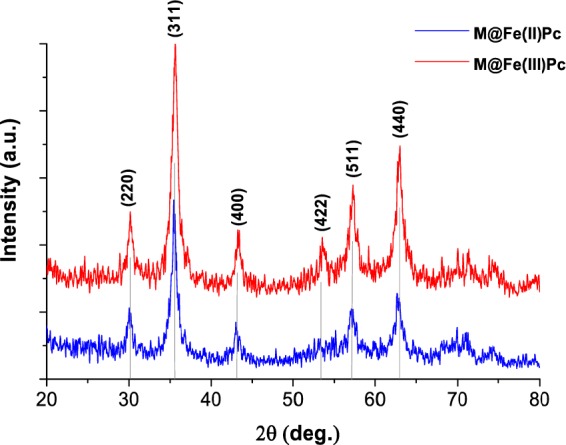
XRD patterns of the synthesized photocatalysts.

The structure phase and average core size of the synthesized catalysts were analyzed based on the recorded XRD patterns of the obtained samples. Figure 2 shows the XRD patterns of M@Fe(II)Pc and M@Fe(III)Pc catalysts. Magnetite (Fe_3_O_4_) was the dominant crystalline phase in all samples exhibiting the typical spinel cubic structure of iron oxide. The peak positions at 2*θ* = 30.245°, 35.603°, 43.242°, 53.663°, 57.113°, and 62.728°, assigned to six indexed planes (2 2 0), (3 1 1), (4 0 0), (4 2 2), (5 1 1) and (4 4 0), respectively, confirm that the obtained nanoparticles consist of crystalline Fe_3_O_4_.

The narrow diffraction peaks of the obtained XRD patterns indicate that all samples consist of nanocrystals with larger average crystallite sizes of ca. 12 nm and 16 nm, for M@Fe(II)Pc, and M@Fe(III)Pc respectively; they were estimated by Scherrer’s equation from the recorded XRD data on each sample (according to the linewidth of the (3 1 1) plane refraction peak):

$${d}_{XRD}=\frac{K\lambda }{\beta \,\cos \,\theta },$$

where *λ* = 0.154 nm is the incident X-ray wavelength, *K* = 0.94 is the particle shape factor for magnetite, *β* is given by the full width at half-maximum of the (3 1 1) diffraction reflection and *θ* is the corresponding diffraction angle (here, 2*θ* = 35.6°). The results indicated that the capped organic layers did not change crystalline phase of Fe_3_O_4_. The XRD data is in agreement with the HR-TEM results.

**Figure 3:**
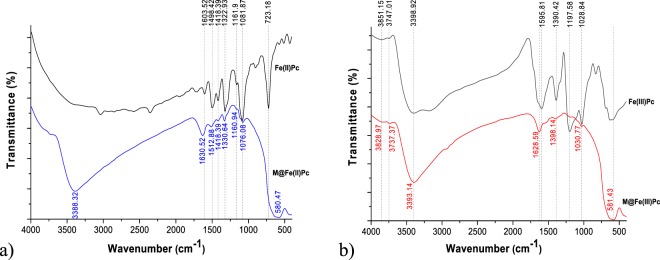
FTIR spectra (**a**,**b**) of the magnetic catalysts M@Fe(II)Pc (left) and M@Fe(III)Pc (right).

Figure 3 shows the FTIR spectra of hybrid magnetic nanoparticles which were analyzed comparatively to the ones of the individual component materials. Literature findings showed that FTIR spectra of magnetite, which exhibits two strong infrared absorption bands at 570 cm^−1^ (υ1) and 390 cm^−1^(υ2), can be assigned to the Fe–O stretching mode of the tetrahedral and octahedral sites for the υ1 band and the Fe–O stretching mode of the octahedral sites for the υ2 band^22^. These main Fe-O vibration bands may differ as function of various factors related to magnetite synthesis protocol, oxidation, stoichiometry of magnetite (Fe(II)/Fe(III) ratios), etc^23–31^. As an example, FTIR investigation of hydrothermally synthetized magnetite showed the two absorption bands at 584 cm^−1^ and 442.03 cm^−1^ respectively^32^. Accounting for the above, the vibration peaks at 580 cm^−1^, observed in both catalysts, are assigned to Fe-O stretching vibration of Fe–O bonds of Fe at tetrahedral and octahedral sites.

The band between 1000 and 1650 cm^−1^ is assigned to the phthalocyanine cycle, and is related to the formation of FePc from the coordination between iron atoms and nitrogen atoms in phthalocyanine ring. The peak at 1161.9 cm^−1^, corresponding to variable C–N stretching^33^ from Fe(II)Pc, but shifted towards lower wavenumbers in the M@ Fe(II)Pc (1160.94 cm^−1^), as well as the peak at 1322.93 cm^−1^ corresponding to N–O symmetric or C–N stretching^33^ in Fe(II)PC, but shifted towards higher wavenumber in the M@ Fe(II)Pc (1330.64 cm^−1^) indicate FePc binding on to magnetite. The peak at 1498.42 cm^−1^ in Fe(II)Pc, corresponding to medium C-C stretching vibrations / strong C=O stretching/C–H stretching, is shown as slightly sifted at 1512.88 cm^−1^ in M@Fe(II)Pc (Table 1). This indicate that one of the above functional groups may contribute to Fe(II)Pc binding onto magnetite surface. The particular peak at 1418.39 cm^−1^ possibly being assigned to C–C stretching or C–H stretching or free O–H bending^33^, it is present in both, Fe(II)Pc and M@Fe(II)Pc spectra, and it seems not be involved in magnetite functioning, as it is not shifted. Specific to Fe(III)Pc are the following vibrations: 1028.84 cm^−1^ and 1390.42 cm^−1^, which are also present in the M@Fe(III)Pc, but slightly shifted toward positive wavenumbers at 1030.77 cm^−1^ and 1398.14 cm^−1^, respectively (Table 1). They indicate S=O stretching^33^ which contribute to Fe(III)Pc binding onto magnetite via sulfoxide groups and sulfate groups, respectively, corresponding to the molecular structure of our initial Fe(III)Pc compound. The particular peak at 1197.58 cm^−1^, present in both Fe(III)Pc and M@Fe(III)Pc spectra, and being assigned to S=O stretching^33^, may belong to another sulfate site, which seems not be involved in magnetite functioning. Vibrations observed in the interval of 1595.81 cm^−1^ and 1630.62 cm^−1^ are present in the both FePc compounds and functionalized magnetite spectra. They can be assigned to C=C stretching vibrations of atoms which are present in aromatic rings of FePc compounds. Moreover, the magnetite-PEG interaction is not evidenced by intense well-defined vibration peaks since the polymeric shell linking the FePc on the magnetic cores is very thin; however, vibration band at 1630 cm^−1^ as well as bands around 1400 and 1600 cm^−1^ might be assigned to the −CH_2_ and −CH_3_ groups and symmetric and asymmetric stretching vibration of carboxyl group, respectively, of the PEG chains^34^. The broad band around 3400 cm^−1^ corresponds to the stretching vibrations of the hydroxyl groups in the materials composition. These results indicate the formation of Fe_3_O_4_/FePc hybrid material.

**Table 1:** Synthetic summary of FTIR data interpretation.

Wavelength, cm^−1^					Bond and vibration type	Reference
Magnetite	Fe(II)Pc	M@Fe(II)Pc	Fe(III)Pc	M@Fe(III)Pc		
581		580.47		581.43	Strong Fe-O stretching	(563)^23^; (573)^24^; (570)^25,30^; (584)^26^; (588)^28,57^
3381		3388.32	3398.92	3393.14	H-O stretching from hydrating water molecules	(3433, 3438, 3452)^24^; (3379)^25^; (3450)^57^; (3438)^58^; (3408)^23^
	723				Strong N-H wog C-N bending	(650–900)^59^ (723)^60^
			1028.84	1030.77	S=O from sulfoxide groups	(1070–1030)^33^
	1161.9	1160.94			Variable C-N stretching strong C-O stretching	(1000–1250)^59^ (1210–1163)^33^
			1197.58	1197.58	Strong S=O stretching from sulphate	(1200–1185)^33^
	1322.93	1330.64			N-O symmetric stretching C-N stretching	(1372–1290)^33^ (1266–1342)^33^, (1000–1250)^59^
			1390.42	1398.14	Strong S=O stretching from sulphate	(1380–1415)^33^
	1418.39	1418.39			Medium C-C stretching C- H stretching H-O bending	(1400–1600)^59^ (1375–1450)^33,59^ (1440–1395)^33^
	1498.42	1512.88			Medium C-C stretching Strong C=O stretching C- H stretching N-O stretching	(1400–1600)^59^ (1630–1760)^59^ (1375–1450)^33,59^ (1500–1550)^33^ (accompanied by peaks at 1290–1372)^33^
	1603.52	1630.62	1595.81	1628.59	C=C stretching	(1600–1650)^33^

The fabricated magnetic catalysts possess high saturation magnetization values as shown in our previous reports studying magnetite-based photocatalysts^35^. The good magnetic properties reveal that the prepared catalysts could be easily separated from reaction medium by the use of external conventional magnet without loss of the magnetic material.

**Figure 4:**
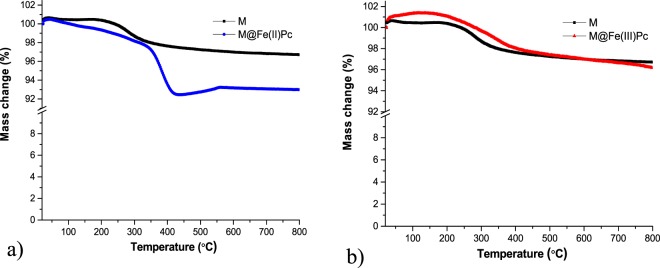
TG curves of the (**a**) M@Fe(II)Pc and (**b**) M@Fe(III)Pc relative to M sample.

The obtained TG curves of the hybrid magnetic photocatalysts are shown in Fig. 4. The results showed the weight loss steps up to 800 °C. The weight loss step over 350 °C was associated with degrading phthalocyanine.

Thus, the total weight loss was about 3.28% for the sample M, 7.01% for the M@Fe(II)Pc catalyst and 3.84% in the case of the M@Fe(III)Pc catalyst. For all the catalysts, the weight loss is including the residual water loss. At temperature above 200 °C the TG curves of the M and M@Fe(III)Pc shows a weight increase of about 0.65%, most probably due to the oxidation of the magnetite into maghemite as previously mentioned^36^. As shown in Fig. 4, the most pronounced mass loss, was occurred for the M@Fe(II)Pc and M@Fe(III)Pc catalysts between 200 °C and 400 °C and it is assigned to the polymer decomposition and burning. The TG curve of the M@Fe(II)Pc present a weight decrease in the temperature range from 100 °C to 400 °C and a mass increase after 400 °C. This behavior, different from the others two samples, can be due to the fact that this sample is covered by a bigger amount of polymer (7.01%), which makes it stable to oxidation over this temperature range. Thus, after the polymer is decomposed, a slight increase (0.5%) in mass occurs, which corresponds to the oxidation of the magnetite to maghemite. The weight loss difference between the M and the M@Fe(II)Pc is about 3.73%, indicating the presence of the Pc(II) polymers in the photocatalyst. In the case of the M@Fe(III)Pc photocatalyst, the difference is only of 0.56%, indicating less polymer covering the magnetite in this sample.

### Kinetics of BPA photodegradation and cytotoxicity of the samples

The effect of catalysts concentration, hydrogen peroxide concentration and the initial concentration of micropollutant, as well as the reutilization of the catalyst have been considered for the photodegradation of BPA in the present study. Additionally, the concentration of singlet oxygen has been determined.

**Figure 5:**
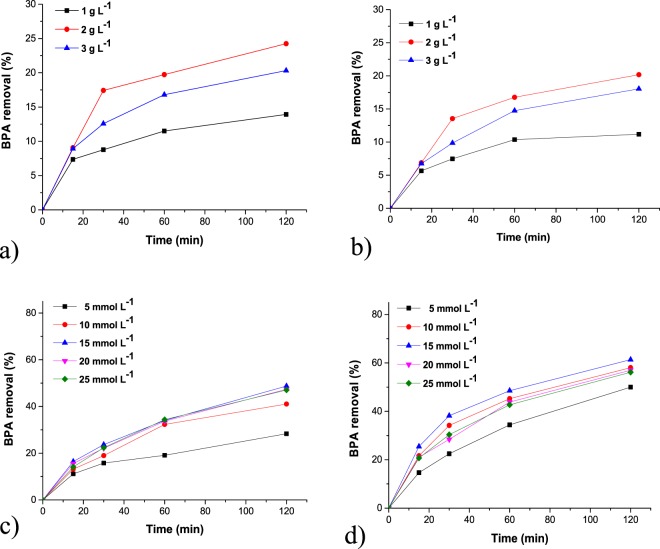
Effect of catalyst concentration ((**a**) M@Fe(II)Pc catalyst; (**b**) M@Fe(III)Pc catalyst) and hydrogen peroxide concentration ((**c**) M@Fe(II)Pc catalyst; (**d**) M@Fe(III)Pc catalyst) on the photodegradation of BPA under UVA light. Initial conditions: 2.0 μmol L^−1^ BPA, 2.0 g L^−1^ of photocatalyst for experiments c,d, pH 6.6, *T* = 25 °C.

Three concentrations of catalyst (1.0, 2.0, 3.0 g L^−1^) were used in our experiments. According to the results presented in Fig. 5a,b, the highest degradation has been achieved at catalyst concentration of 2 g L^−1^ for both catalysts, slightly more active being the M@Fe(II)Pc catalyst. The photodegradation of BPA in the presence of both catalysts is between 19 and 28%, depending on the concentration of catalysts used in the experiments.

At higher concentration of the catalyst (>2 g L^−1^) no improvement in the degradation of the pollutant could be observed. This could be explained by the agglomeration of magnetic nanoparticles, at higher concentration. Thus, the concentration of 2 g L^−1^ has been selected for the next experiments.

**Figure 6:**
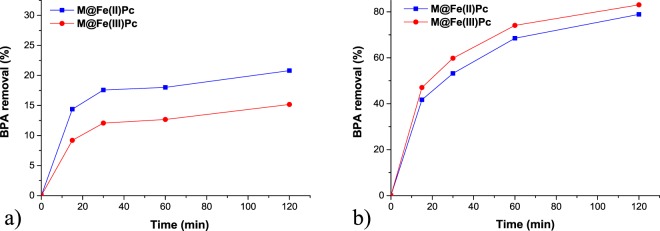
Photodegradation of BPA under natural solar light in the absence (**a**) and in the presence (**b**) of 15 mmol L^−1^ hydrogen peroxide for the two photocatalysts. Initial conditions: 2.0 μmol L^−1^ BPA, 2.0 g L^−1^ of photocatalyst, pH 6.6, *T* = 25 °C.

Under natural solar light the degradation of BPA was between 15 and 20% that is similar to degradation under UVA light (Fig. 6). In the presence of H_2_O_2_ the degradation was enhanced. 83% of BPA in solution was removed by the Fe(III)Pc photocatalyst during 120 minutes of irradiation.

**Figure 7:**
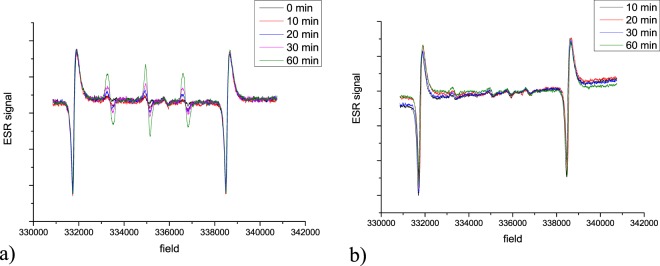
ESR signal of TMP-OH in the presence of M@Fe(II)Pc (**a**) and M@Fe(III)Pc (**b**) catalysts under UVA irradiation. Initial conditions: 1.0 g L^−1^ photocatalyst, photon flow of 9.64 × 10^−8^ Einstein s^−1^, irradiation depth of 1.2 cm, irradiated surface of 5.72 cm^2^, room temperature.

Additionally, the property as a photosensitizer of the catalysts to generate singlet oxygen was investigated. The results are presented in Fig. 7. and show that, after 60 minutes of irradiation, in the presence of M@Fe(II)Pc catalyst, 9.8 µmol L^−1^ of singlet oxygen was generated, whereas in the presence of M@Fe(III)Pc catalyst only 1.26 µmol L^−1^ were produced. This indicate that the M@Fe(II)Pc catalyst can promote more efficiently the formation of ^1^O_2_, which enhances the degradation of BPA.

The proposed mechanism could be as follows: the BPA oxidation and degradation is triggered by the Reactive Oxygen Species (ROSs) generated by a molecular oxygen activation process which occurred in the presence of photosensitizer under the UV irradiation^5,35,37–41^. Detailing, a photon is adsorbed by an electronically excited singlet state which can further undergo intersystem crossing and generate longer lived excited triplet state, promoting a sensitizer. More singlet oxygen is then being produced by energy transfer towards dissolved molecular oxygen. The magnetic core generates electrons and holes in the valence band which contribute to Fenton photocatalysis.

The addition of hydrogen peroxide improved the degradation of BPA. Five concentrations of H_2_O_2_ have been selected (Fig. 5c,d). The M@Fe(III)Pc catalyst was more efficient. In 120 min using 15 mmol L^−1^ H_2_O_2_, 67% of BPA has been decomposed. At the same concentration of hydrogen peroxide the degradation of BPA was increased to 82% and to 70.46% in the presence of M@Fe(III)Pc and M@Fe(II)Pc, respectively, after 240 min. By increasing of hydrogen peroxide concentration to 20–25 mmol L^−1^ no improvement of the decomposition of the micropollutant could be observed. This could be because the generated hydroxyl radicals produced hydroperoxyl radicals (HO_2_•) in the presence of a local excess of H_2_O_2_. The hydroperoxyl radicals are much less reactive and do not contribute to the oxidative degradation of the organic substrate which occurs only by reaction with HO•. Similar behaviour shows the M@Fe(II)Pc catalyst. Therefore, for our further experiments a concentration of 15 mmol L^−1^ H_2_O_2_ was chosen. No significant photodegradation of BPA occurred in the presence of H_2_O_2_ alone (in the absence of the photocatalysts). High catalytic removals of 0.02 mmol L^−1^ of RB195 and RhB dyes (ca. 80%) from aqueous solution were also achieved by Han *et al*.^42,43^ in the presence of FePc and CuFePc immobilized on PAN, 2.50 mmol L^−1^ H_2_O_2_ and pH 6. Literature studies showed that phenol has been efficiently degraded by photocatalytic activity of iron(II/III) phthalocyanine supported on graphene^44^ or zeolites^45^. They indicated that the photocatalytic activity is improved by the π–π stacking interaction when FePC is loaded on graphene, and also by the addition of the oxidant^46^. The mechanism of degradation can be attributed to photo-Fenton-like processes and is clear presented by Norman^46^ and other authors^44,47–50^ as follows: in a Fenton process, the oxidation process being carried out by the hydroxyl radicals directly produced from the reaction between H_2_O_2_, Fe^2+^ or Fe^3+^ at the catalyst surface. Hydroxyl radicals are considered the most reactive species responsible for degradation of organic pollutants^46^. On the surface of solid catalyst the hydroxyl radicals are generated by complexation mechanism^48^. According to Rodriquez *et al*.^47^ these reactions are:

$${{\rm{F}}{\rm{e}}}^{3+}+{{\rm{H}}}_{2}{{\rm{O}}}_{2}\to {{\rm{F}}{\rm{e}}}^{2+}+{{\rm{H}}}^{+}+{{\rm{H}}{\rm{O}}}_{2}\bullet $$

$${{\rm{Fe}}}^{3+}+{{\rm{HO}}}_{2\bullet }\to {{\rm{Fe}}}^{2+}+{{\rm{H}}}^{+}+{{\rm{O}}}_{2}$$

$${{\rm{H}}{\rm{O}}}_{2\bullet }+{{\rm{H}}}_{2}{{\rm{O}}}_{2}\to {{\rm{H}}}_{2}{\rm{O}}+{{\rm{O}}}_{2}+{\rm{O}}{\rm{H}}\bullet $$

– in reaction with FePC and UV irradiation^44^:

$${{\rm{Fe}}}^{3+}+{{\rm{H}}}_{2}{{\rm{O}}}_{2}+{\rm{hv}}\to {{\rm{Fe}}}^{2+}+{\rm{OH}}\bullet +{{\rm{H}}}^{+}$$

The metal phthalocyanine is photosensitized also under UV irradiation^44^:

$${\rm{FePC}}+{\rm{hv}}\to {{\rm{FePC}}}^{\ast }\to {{\rm{FePC}}}^{+}+{e}^{-}$$

$${{\rm{e}}}^{-}+{{\rm{H}}}_{2}{{\rm{O}}}_{2}\to {\rm{O}}{\rm{H}}\bullet +{{\rm{O}}{\rm{H}}}^{-}$$

As shown by Wu *et al*. and Dai *et al*.^49,50^, the conversion of Fe(III) to Fe(II) have been probably promoted by FePc. The coordination number of Fe site in FePc structure it is changed by its binding with the hydroxyl radical (as axial ligand). This leads to geometric and electronic structural variations within the FePc^27^, which enhance fast electron transfer for the Fe(II)/Fe(III) cycle, generates unsaturated sites for oxidants and favour potential coordination with new Fe centers^51^.

**Figure 8:**
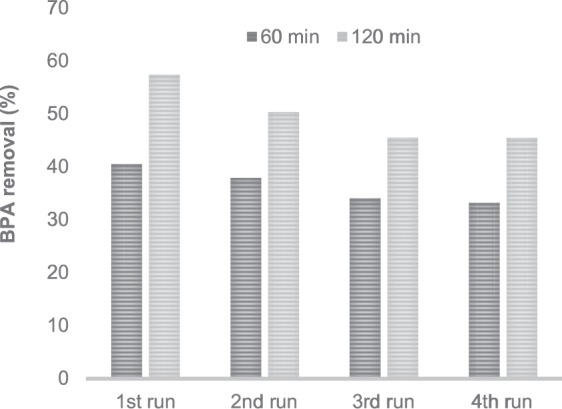
Reutilization of M@Fe(III)Pc catalyst over four cycles for the photodegradation of BPA. Initial conditions: 2.0 μmol L^−1^ BPA, 2.0 g L^−1^ of photocatalyst, pH 6.6, *T* = 25 °C.

The reutilisation of the catalysts is very important for their application. The M/PEG @Fe(III)-Pc catalyst has been chosen for reutilisation experiments (Fig. 8).

The activity of the catalyst remains good over four runs. Moreover, no Fe leaching was detected during the degradation experiments. In the paper of Han *et al*.^51^, the removal rate of RR195 in FePc-PAN/PMS system remained similar in five successive runs, indicating the good reusability of FePc-PAN for PMS activation under visible light irradiation. In another study^43^, the authors found that due to the inhibition of the light absorption by intermediate accumulation on the surface of CuFePc-PAN, a 12% decline of RhB removal was observed after five running cycles. In the study of Ouedraogo *et al*.^52^ the percentage of Orange II removal only slightly decreases from 95.8 to 91.2% after the fifth cycle. Our results are in good agreement with those reported in the literature so far.

**Figure 9:**
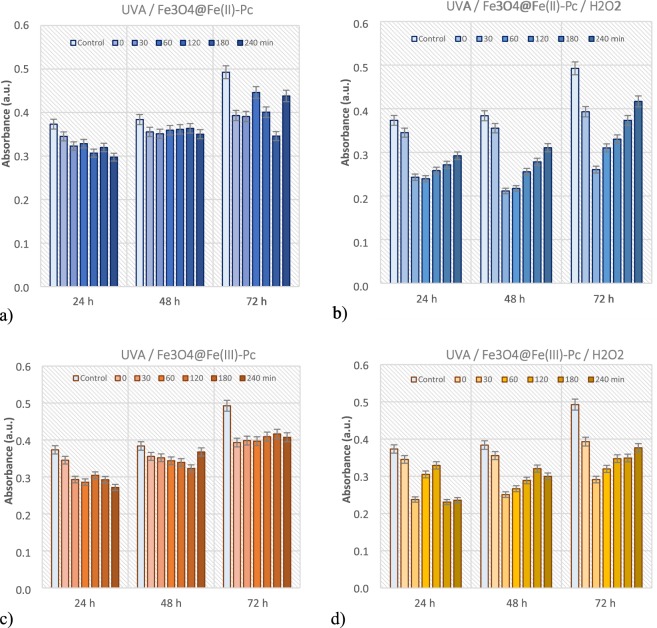
*MCF-7* cell viability assay for the photodegradtion of BPA over both catalysts in the absence (**a**,**c**) and presence of hydrogen peroxide (**b**,**d**). Initial conditions: 2.0 μmol L^−1^ BPA, 2.0 g L^−1^ of photocatalyst, 15 mmol L^−1^ H_2_O_2_, pH 6.6, *T* = 25 °C.

The potential toxicity of the degradation products in the reaction medium is important issue for each wastewater treatment. To exhibit cytotoxic effects after the proposed treatment, the *MCF-7* cell viability assay was carryed out (Fig. 9).

The cell viability results showed that the cytotoxicity of metabolically active cells slightly increased over the studied incubation time, indicating that after treatment the samples are less cytotoxic. Compared to control, the low absorbance could be associated with the intermediates that have been detected to exhibit cytotoxic effect.

## Conclusion

In this study two types of iron phthalocyanine (FePc)-sensitized magnetic nanocatalysts were investigated for the photodegradation of BPA, an endocrine disruptive chemical (EDC), that is able to induce damages in living organisms. The quasi-spherical shape of the nanoparticles, with diameters up to 15 nm, were identified by HR-TEM. The ultrathin coating of phthalocyanine ring of ca 1 nm surrounding the magnetic cores was also evidenced and confirmed by FTIR spectra and TG analysis. XRD patterns of the obtained samples confirmed that magnetite was the dominant crystalline phase in all samples. In the presence of M@Fe(II)Pc catalyst, 9.8 µmol L^−1^ of singlet oxygen was generated after 60 minutes of irradiation, whereas in the presence of M@Fe(III)Pc catalyst only 1.26 µmol L^−1^ were produced. The optimum concentrations of catalyst and hydrogen peroxide were 2.0 g L^−1^ and 15 mmol L^−1^ H_2_O_2_, respectively. The photodegradation of BPA in the presence of both catalysts was between 19 and 28%, whereas, with the addition of H_2_O_2_, the BPA removal have increased (between 49 and 83%) and it was accelerated by exposure to natural solar light. In the presence of hydrogen peroxide, the M@Fe(III)Pc catalyst was more active. The activity of both catalysts remains good over four consecutive runs. The *MCF-7* cell viability assay showed that, after treatment, the water samples were less cytotoxic. Our results showed that both catalysts can be successfully applied in wastewater treatment.

## Methods

### Materials and characterization methods

All aqueous solutions were prepared with fresh ultrapure water (0.055 µS cm^−1^) produced by an Evoqua LABOSTAR Pro TWF UV unit. High purity chemicals (SIGMA-ALDRICH) were used in all experimental procedures. Iron(II) phthalocyanine (Fe(II)Pc) and iron(III) phthalocyanine-4,4′,4′′,4′′′-tetrasulfonic acid with oxygen monosodium salt hydrate (Fe(III)Pc) were chosen as sensitizers and were immobilized on magnetic nanoparticles in order to catalyse the photooxidation of BPA in solution.

A BANDELINE SONOPULS homogenizing system, model HD4100, equipped with a HF generator GM 4100, ultrasonic converter UW100, standard horn SH 100 G and a 13 mm titanium probe, was set-up at 20 W for the sonochemical synthesis of the magnetic nanoparticles.

The synthesis of the photocatalysts was followed by the characterization of their physico-chemical properties. Ultra-high resolution transmission electron microscopy (UHR-TEM) images were acquired by a LIBRA200MC/CARL ZEISS GmbH instrument (Germany). X-ray diffraction (XRD) patterns were recorded by a BRUKER AXS D8-Advance X-ray diffractometer (Germany) using Bragg-Brentano configuration and the Cu-Kα radiation (*λ* = 0.154 nm); the *DIFFRAC*^*plus*^ *Eva* software was also used in order to estimate the average crystallite size in the synthesized nanomaterials based on Scherrer’s equation. Standard Si material and its spectra was used as a reference material for FWHM analyses and calculation of relative particle size. The immobilization of phthalocyanines on the magnetic cores was evaluated by Fourier transform infrared (FT-IR) spectroscopy (JASCO 6100 spectrometer) at room temperature; powder samples prepared in KBr discs were used to record the FT-IR spectra between 400 and 4000 cm^−1^, with a resolution of 4 cm^−1^. Thermogravimetric analyses (TGA) were carried out in argon atmosphere using a NETZSCH STA 409 PC/PG device, in the temperature range from 20 °C to 800 °C with a heating rate of 5 K min^−1^. Electron spin resonance (ESR) spectra were recorded using a MINISCOPE MS300 device (Magnettech GmbH, Berlin, Germany) and the diamagnetic 2,2,6,6-tetramethyl-4-piperidinol (TMP-OH, 40 mmol L^−1^) as trap for the molecular singlet oxygen (^1^O_2_) generated in solution in the presence of the photocatalysts; the entire settings and method is described in detail in our previous work^35^.

### Photocatalyst synthesis

The preparation of the two photocatalysts consisted in a multi-step procedure, starting with the sonochemical synthesis of the magnetite (Fe_3_O_4_) cores and their subsequent stabilization with low molecular polyethylene glycol (PEG), immediately followed by the sensitizing of the obtained nanoparticles with photoactive compounds, namely iron phthalocyanines (FePc). The detailed procedure was described in our recent study^35^. Briefly, in here, the conventional wet co-precipitation route, a very efficient method for Fe_3_O_4_ nanoparticles synthesis, was carried out under ultrasound irradiation, maintaining a constant temperature of 60 °C, and normal atmospheric conditions. The as-synthesized bare magnetic nanoparticles were then rapidly functionalized with PEG in order to prevent their agglomeration and oxidation, yielding Fe_3_O_4_/PEG nanoparticles, further denoted as M. Equal amounts from the fresh black slurry M were further functionalized with either Fe(II)Pc or Fe(III)Pc. A volume of 20 mL of 3.72 mmol L^−1^ solution of each iron phthalocyanine was gradually poured onto 0.5 g of M and kept under rapid continuous mechanical stirring and heating (70 °C) for 210 minutes. The resulted magnetic photocatalysts, M@Fe(II)Pc and M@Fe(III)Pc respectively, were finally purified (by repeated rinsing with ultrapure water and absolute ethanol alternated with magnetic decantation for removing the residual products) then dried in an oven under vacuum at 60 °C.

### Catalytic activity

Kinetic experiments were carried out at laboratory-scale, in mild conditions (i.e. initial near-neutral pH of 6.6, room temperature 23 ± 2 °C) in order to assess the catalytic activity of the FePc-based photocatalysts for the BPA degradation in aqueous solution. A solution of 2 µmol L^−1^ BPA was used as initial concentration. Before starting the kinetic experiments, the catalysts was tested in the dark in the presence of BPA; the solution was analyzed at regular time intervals indicating that no adsorption phenomena occurred. Next, the photooxidation tests were carried out in 50-mL Berzelius glasses, each one containing 5 mL pollutant solution, placed on a cooled support and under a UVA light source (Analytik Jena bench lamp, *λ* = 365 nm, 15 W) consisting in two parallel self-filtering, low pressure ‘black light’ tubes; the diameter of the irradiated surface area was 11.34 cm^2^, the irradiation path-length was 0.8 cm, and photon flow of 9.28 × 10^−8^ Einstein s^−1^ (measured by polychromatic actinometry with phenylglyoxylic acid in AcN:H_2_O = 3:1 v/v)^53^. In the case of natural solar light exposure experiments, the same protocol was followed the diameter of the irradiated surface area was the same and photon flow was 9.67 × 10^−8^ Einstein s^−1^. The experiments were performed in July 2019, Iasi (47° 9′ 6.2136″ N, 27° 35′ 16.4904″ E), Romania.

The catalytic performance of the synthesized photocatalysts was assessed by evaluating the effect of catalyst concentration, hydrogen peroxide dosage, under light exposure for a total reaction time of 120 min. Catalyst concentration of 1.0, 2.0 and 3.0 g L^−1^ were tested in the BPA degradation experiments at various time intervals with in the total reaction time of 120 min. Five H_2_O_2_ concentrations, 5, 10, 15, 20 and 25 mmol L^−1^, freshly prepared from a 30% H_2_O_2_ standard solution, were studied in the next step. During kinetic experiments, aliquots of 300 μl of BPA solution were withdrawn from the reaction vessels at different time intervals (15, 30, 60 and 120 min), immediately filtered through a 0.22 µm Millipore nitro-cellulose membrane. The concentration of BPA in the supernatant solutions was determined by a high performance liquid chromatograph (HPLC-UV-VIS/DAD Agilent Technologies 1260 Infinity series). The description of experimental set-up and detailed method of analyses can be found in our previous study^54^. The experiments were performed in duplicate, the calculated relative standard error being under 3%.

For determination of the dissolved iron, the Ferrozine method was used^55^.

The detailed procedure of the *MCF-7* cell viability assay, performed on the studied samples in order to test their cytotoxicity level, is described in our earlier publications^37,56^.

## Data Availability

All data generated or analysed during this study are included in this published article.
